# Investigation of observational methods assessing workload of static working postures based on surface electromyography

**DOI:** 10.3233/WOR-192854

**Published:** 2019-02-27

**Authors:** Tobias Hellig, Vera Rick, Alexander Mertens, Verena Nitsch, Christopher Brandl

**Affiliations:** RWTH Aachen University, Institute of Industrial Engineering and Ergonomics, Aachen, Germany

**Keywords:** Postural load, exposure assessment, musculoskeletal disorders

## Abstract

**BACKGROUND:**

A large number of different methods are available to identify and assess working postures. Although observation-based methods are most commonly used in practise, investigations showed different results regarding validity of such methods.

**OBJECTIVE:**

To investigate validity of one of the most commonly used observation-based assessment method in ergonomics, the Ovako Working Posture Analysing System (OWAS) and the European standard EN 1005-4 for evaluation of working postures, an experimental laboratory study was conducted.

**METHODS:**

Muscle activity was measured under combinations of static working postures of trunk inclination and shoulder flexion to compare these measurements and observation-based assessments according to OWAS and EN 1005-4. In order to investigate the magnitude of correspondence between muscle activity and observation-based assessments, Spearman rank correlation coefficients (r_s_) were calculated.

**RESULTS:**

Significant correlations were found between OWAS and muscle activity (range from r_s_^2^ = 0.17 r_s_^2^ = 0.55). Significant correlations were found between EN 1005-4 and muscle activity (range from r_s_^2^ = 0.34 to r_s_^2^ = 0.74).

**CONCLUSIONS:**

Results emphasise a need for further developments of observation-based methods, since the two investigated methods showed a variance of validity ranging from small to large. Such improvements may also form a better basis for the ergonomic improvement of working conditions in practise, which is highly necessary due to a constantly high prevalence of MSDs in the last decades.

## 1. Introduction

Musculoskeletal disorders (MSD) are a common problem in many industrialized countries [1]. In Germany and other industrialized countries MSDs are the main reason for health-related sick leave and early retirement [2]. Therefore, MSDs are responsible for tremendous financial and social costs [3], which have even increased in recent years [4].

Investigations have identified external exposure factors such as working posture, force and repetitiveness to determine workload and to be responsible for the development of MSDs [5]. Among these factors, working posture is the most important factor related to the development of MSDs [1, 6]. A wide range of assessment methods has been developed to quantify workload as a function of external factors [7, 8] or to assess workers’ response to workload [6]. These methods can be classified by their data collection strategy into subjective, objective and observation-based methods [5]. Subjective methods like interviews and questionnaires can be used to collect data on physical and psychosocial factors of exposure. Overall, such methods show low validity regarding the assessment of workload [9, 10]. Objective methods are able to capture highly accurate data on a wide range of exposure variables such as joint motion or posture angles as well as response variables such as muscle activity or muscle fatigue [11]. However, the use of objective methods is expensive and a considerable effort of sampling and post-processing data is required. Observation-based methods like Ovako Working Posture Analysing System (OWAS) [12], Rapid Upper Limb Assessment (RULA) [13], Rapid Entire Body Assessment (REBA) [14] or EN 1005-4 have been developed to identify and assess exposure to workload by systematic observations of various exposure factors, i.e. working postures. Each one of these methods entails both advantages and disadvantages. The use of observation-based methods is inexpensive and can be done without any interruption of the work process [9]. However, some studies revealed only a moderate-to-good validity of observation-based methods regarding the assessment of macro-postures, i.e. trunk inclination or shoulder movement [9, 11]. Observation-based assessment methods show lower validity regarding the assessment of micro-postures, i.e. finger or hand movements [11]. Furthermore, most of observation-based assessment methods do not consider interaction effects of exposure variables [9]. Nevertheless, interaction effects of exposure variables have been shown to influence workload significantly [15]. Also, previous work of the authors reported significant interaction effects of working postures [16]. Therefore, quantitative assessment procedures of observation-based methods are hypothetical in some cases. Different studies investigated objectivity, reliability and validity of observation-based methods, systematic reviews of observation-based methods can be found at Denis et al. [17], David [10] and Takala et al. [11]. OWAS, one of the main-representative observation-based methods, which is most often used in practise [18], was investigated with regard to validity by various studies. A good association between subjective discomfort ratings and OWAS ratings could be observed by Kayis and Kothiyal [19] and Olendorf and Drury [20]. Low association was found between OWAS and continuous measurement of trunk bending by Burdorf et al. [21]. A comparison of assessment results of OWAS and the revised NIOSH lifting equation revealed great differences [22]. In order to evaluate validity of observation-based methods, it should be noted that there is a missing gold-standard for the observation-based assessment of external exposure factors [10, 11, 17]. For this reason, it seems promising to investigate validity of observation-based assessment results by a comparison with measurements of response variables [23]. For this kind of investigation, the stress-strain concept provides a theoretical framework: In the context of the stress-strain concept [24], an objective evaluation of workload is conducted by the measurement of appropriate physiological response variables. The selection of response variables depends on the one hand on the kind of workload and on the other hand on the limiting factor of the human organism during exposure to the kind of workload [25]. Especially during static working postures, which is one major risk factor for the development of MSDs, muscle activity represents the limiting factor of the human body since the internal muscle pressure causes an interruption of blood flow and thus an oxygen shortage of the muscle occurs [26]. Therefore, an investigation of static working postures has to be based on the measurement of muscle activity [27].

To investigate validity of observation-based assessment results, a laboratory experiment was conducted. Muscle activity was measured under combinations of static working postures of trunk inclination and shoulder flexion to compare objective measurements and observation-based assessments according to OWAS and EN 1005-4. OWAS was chosen as a main representative of observation based methods most often used in practise [18]. EN 1005-4 was chosen as representative method of the state of the art which is based on the relevant findings of scientific research.

We hypothesised a significant increase of muscle activity with an increasing deviation of postures from neutral postures. Furthermore, a significant interaction effect of different posture factors, i.e. trunk inclination and shoulder flexion, is hypothesised. Furthermore, a significant linear association between the results of OWAS, EN 1005-4 and muscle activity is hypothesised.

## 2. Method

### 2.1. Participants

A sample of 24 volunteers (11 female) aged between 20 and 28 (M = 24.6, SD = 1.99) participated in this study. The sample was not gender-balanced, since normalised muscle activity values are independent from gender [28]. Persons were excluded from this study if they reported symptoms of musculoskeletal injuries during the last twelve months. Informed written consent was obtained prior to participating in this study.

### 2.2. Experimental design

**Fig.1:**
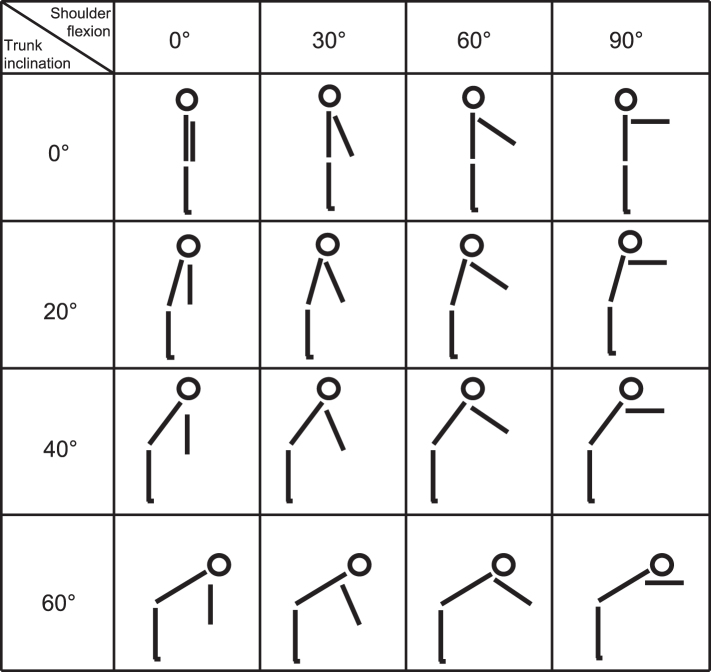
Different types of investigated working postures.

In a standing position, participants bent their trunk and flexed both arms in four different angles. Each angle of the trunk was combined with each angle of the arms, whereby trunk inclination was varied in steps of +20° and shoulder flexion was varied in steps of +30°. This resulted in 16 different postures (see Fig. 1). Each posture was held for one minute. The investigated working postures were chosen to cover the entire range of motion of trunk inclination and shoulder flexion. The period of one minute static working posture was determined to ensure practical relevance and to enable an appropriate total time of the experiment.

During the contraction phase, muscle activity of the eight following muscles was measured by surface electromyography (EMG): left and right trapezius pars descendens (LUT, RUT), left and right trapezius pars ascendens (LLT, RLT), left and right anterior deltoideus (LAD, RAD) and left and right erector spinae (LES, RES). The selection of these muscles was based on their activity as shoulder stabiliser, arm raiser and trunk extensor [29–31]. In order to eliminate influence of muscle fatigue, a one minute resting period was held after each working posture, and a five minute resting period was held after five working postures. The length of time of resting periods was based on evaluations in previous studies conducted by the authors [16]. The order of the test conditions was systematically varied according to Latin square design.

### 2.3. Procedure

The experimental procedure was divided into two main parts: (1) preparation of test persons and (2) investigation of workload of 16 different static working postures.

In the first part of the experimental procedure, the sensor and electrode placement was conducted according to the European Recommendations for Surface Electromyography (SENIAM) [30]. Since amplitudes of EMG measurements show high differences between and within test persons, comparisons of different measurements are enabled by normalization to a reference contraction [32, 33]. Therefore, the highest voluntary electrical muscle activity during a variety of postures was obtained, which is the maximal voluntary contraction (MVC) [34]. Generally, the muscle activity during the experimental conditions is expressed as a percentage of the MVC value. In the second part of the experimental procedure, test persons held the 16 different working postures. Continuous EMG signals were captured during the entire contraction phase. During the one minute static contraction phase, working postures were monitored by a software tool to measure trunk inclination angle and shoulder flexion angle by Microsoft Kinect V2. Because it is impossible to maintain real static postures and there are always minimal movements, a deviation of the target working posture of±5° has been accepted during the experimental procedure. The value of±5° is based on results of a study conducted by the authors [16]. If participants have exceeded the range of±5°, they were advised to adjust their posture. During resting periods, test persons were free to stand or sit down. Reliability of posture measurements by Microsoft Kinect V2 has been investigated for example by Patrizi et al. and Braganca et al. showing good accuracy [35, 36].

### 2.4. Data recording and processing

#### 2.4.1. Surface electromyography

A surface electromyography device (Desktop DTS Receiver, Noraxon, Scottsdale, AZ, USA) was used to capture muscle activity. Ag/AgCl self-adhesive 8-shaped dual electrodes (4×2.2 cm; diameter of adhesives: 1 cm; inter-electrode distance: 1.75 cm) were applicate on the skin according to SENIAM standards [30]. Signals were amplified with a gain of 1000 V/V, input impedance of 100 M*Ω* and a common mode rejection ratio of 100 dB. Continuous EMG signals were recorded during muscle contractions with a sampling frequency of 1500 Hz and digitally band-pass filtered (10–500 Hz) with a first-order high-pass filter. The biomechanical analysis software MyoResearch 3.8 (Noraxon, Scottsdale, AZ, USA) was used. Root mean square (RMS) amplitude was calculated with an overlapping moving window of 100 ms. In order to compare EMG data of different conditions of working postures, RMS values of each experimental condition were normalized relative to the values of initial MVC based on equation [1]:

(1) $$\%\mathrm{MVC}\;=\;\frac{\mathrm{RMS}}{\mathrm{MVC}}\;\times \;100\%$$

To minimize the effects of initial movements of the body parts, capturing of EMG signals was started once a test person had assumed the working posture.

#### 2.4.2. Observation-based assessment of working postures

In addition to the objective measurement of muscle activity, the 16 working postures were evaluated using OWAS and EN 1005-4. OWAS assigns one of four action categories (AC) to each working posture. AC 1 characterizes a working posture of minimal exposure level. In ascending order, AC 2 to AC 4 represent an increasing exposure level and increasing risk of musculoskeletal injury.

EN 1005-4 only evaluates postures of single body parts e.g. bending trunk forward is evaluated independent from postures of arms and legs. EN 1005-4 assigns one of four zones to each posture of the trunk as well as to each posture of the arms. Analogous to OWAS ACs the EN 1005-4 Zone 1 characterises a posture of an acceptable exposure level. Zone 2 to Zone 3 characterise an increasing exposure level from conditionally acceptable to not acceptable.

#### 2.4.3. Data analysis

Statistical analyses were conducted using IBM SPSS Statistics 25. A repeated-measures analysis of variance (MANOVA) was used to derive the influence of trunk inclination and shoulder flexion on muscle activity. Since observation-based assessment results are ordinal data, Spearman rank correlation coefficients (r_s_) were calculated to investigate the magnitude of correspondence between objective measurements of muscle activity and observation based assessment results. According to Cohen [37] correlation coefficients were interpreted as trivial (<0.1), small (0.1–<0.3), medium (0.3–<0.5) and large (≥0.5). Significance of all statistical tests was accepted at the *α*-level of *p* <  0.05.

## 3. Results

### 3.1. EMG amplitude and repeated-measures MANOVA

**Fig.2:**
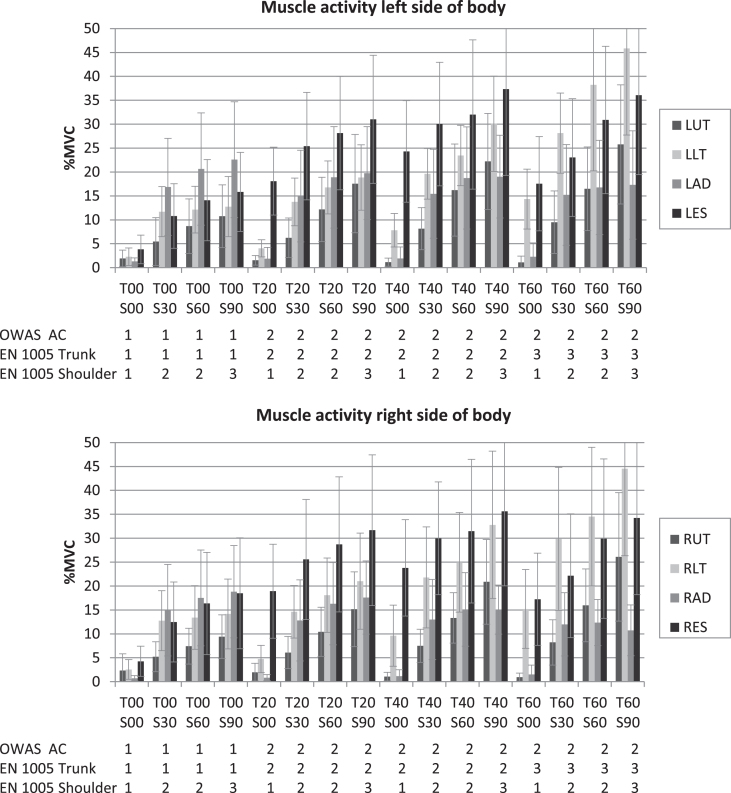
Averaged muscle activity according to ventral trunk inclination angle (T) and shoulder flexion angle (S), assessment results of OWAS and EN 1005-4, error bars indicating standard deviation.

The exercise conditions resulted in considerably different degrees of muscle activity. Figure 2 contains averaged normalized muscle activity of the muscles in the left and right side of the body. As evident from Fig. 2, muscle activity of the investigated muscles (LUT, RUT, LLT, RLT, LAD, RAD, LES, RES) increases with increasing trunk inclination angles and increasing shoulder flexion angles. Overall an increasing shoulder flexion angle and trunk inclination angle increase muscle activity of upper trapezius muscles, lower trapezius muscles and erector spinae muscles. An increase in shoulder flexion angle increases muscle activity of anterior deltoid muscles, whereas a decreasing muscle activity of anterior deltoid muscles could be recognized as a consequence of an increasing trunk inclination angle.

### 3.2. Observation-based assessment results

Results of observation-based assessments are shown with corresponding mean values of muscle activity in Fig. 2. Among the investigated working postures, there are four with an OWAS AC 1 and twelve with an AC 2. Only postures of a trunk inclination angle of 0° are assigned to OWAS AC 1.

The investigated working postures are assigned to two different Zones of EN 1005-4. Postures of trunk inclination angles of 0° are assigned to Zone 1, trunk inclination angles of 20° and 40° are assigned to Zone 2, trunk inclination angles of 60° are assigned to Zone 3. Shoulder flexion angles of 0° are assigned to Zone 1, shoulder flexion angles of 30° and 60° are assigned to Zone 2 and shoulder flexion angles of 90° are assigned to Zone 3.

### 3.3. Correspondence between objective measurements and observation based assessment results

**Fig.3:**
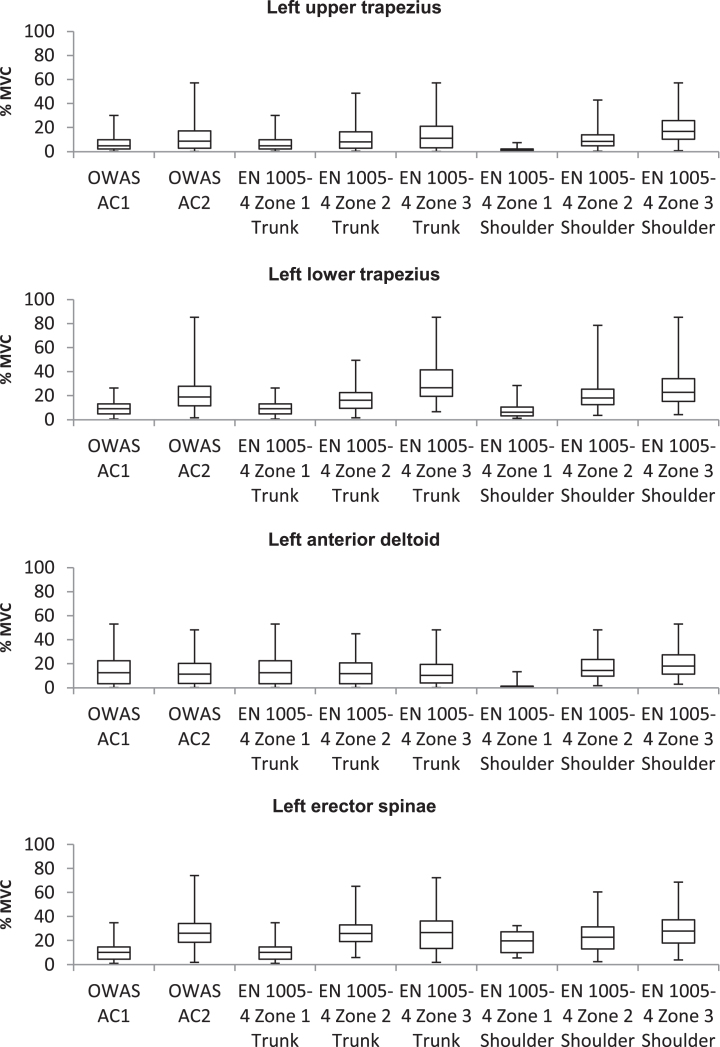
Relationship of left body side muscle activity and assessment results of OWAS and EN 1005-4.

**Fig.4:**
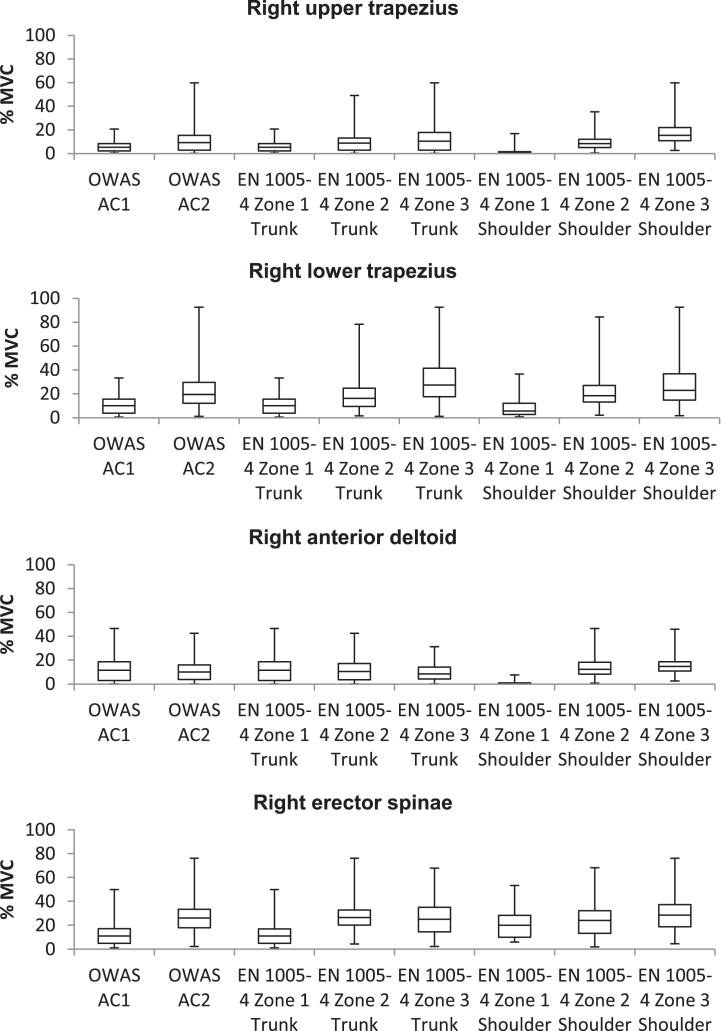
Relationship of right body side muscle activity and assessment results of OWAS and EN 1005-4.

In order to investigate the magnitude of correspondence between muscle activity and OWAS ACs as well as EN 1005-4 Zones, Spearman rank correlation coefficients (r_s_^2^) were calculated. Detailed results are given in Table 1, Figs. 3 and  4. There were significant positive correlations between OWAS ACs and muscle activity of all muscles except for anterior deltoid muscles. Correlation between muscle activity of erector spinae muscles and lower trapezius muscles and shoulder assessment of EN 1005-4 were significant positive. A significant negative correlation was found between shoulder assessment of EN 1005-4 and muscle activity of right anterior deltoid. Furthermore, there were significant positive correlations between trunk assessment of EN 1005-4 and muscle activity of all muscles.

**Table 1:** Results of Spearman’s rank correlation analysis

Muscle	OWAS		EN 1005-4 Trunk		EN 1005-4 Shoulder	
	r^2^	*p*	r^2^	*p*	r^2^	*p*
LUT	0.178	<0.001	0.180	0.180	0.729	<0.001
RUT	0.184	<0.001	0.191	0.191	0.745	<0.001
LLT	0.426	<0.001	0.544	<0.001	0.557	<0.001
RLT	0.391	<0.001	0.496	<0.001	0.536	<0.001
LAD	–0.052	0.312	–0.066	0.196	0.639	<0.001
RAD	–0.070	0.171	–0.109	0.033	0.648	<0.001
LES	0.551	<0.001	0.423	<0.001	0.336	<0.001
RES	0.472	<0.001	0.343	<0.001	0.340	<0.001

Note: LUT = Left upper trapezius; RUT = Right upper trapezius; LLT = left lower trapezius; RLT = right lower trapezius; LAD = left anterior deltoideus; RAD = right anterior deltoideus; LES = left erector spinae; RES = right erector spinae.

### 3.4. Repeated measures MANOVA

Repeated-measure MANOVA was employed to estimate effects of trunk inclination angle and shoulder flexion angel on muscle activity. Trunk inclination angle was found to have a significant effect on muscle activity, V = 1.31, F(21, 195) = 7.20, *p* <  0.001. Shoulder flexion angle was found to have a significant effect on muscle activity, V = 1.44, F(21, 195) = 8.57, *p* <  0.001. Trunk inclination angle and shoulder flexion angle were found have a significant interaction effect on muscle activity, V = 1.00, F(72, 1656) = 3.47, *p* <  0.001.

## 4. Discussion

The aim of the paper was to investigate validity of observation-based assessment results of working postures. Therefore, muscle activity was measured under combinations of trunk inclination and shoulder flexion and compared to observation-based assessment results.

### 4.1. Comparison of EMG, OWAS and EN 1005-4

To investigate validity of observation-based assessment results a correlation analysis according to Spearman was conducted. Calculating a synthetic risk score of a working posture which is associated with the workload brought about by this working posture and the musculoskeletal injury risk resulting of this working posture is a common practise of observation-based methods. The procedure of such observation-based methods depends on different input values characterising the load, for example posture angles or force levels, which are converted by the use of simple equations or tables to a value, which describes the risk of the load [6]. The OWAS ACs and Zones of EN 1005-4 are determined accordingly. Therefore it is to be expected that working postures with higher risk scores cause higher values of muscle activity. In line with this assumption, significant correlations between muscle activity and OWAS ACs were found. A large significant correlation was found between muscle activity of the ES muscles and OWAS ACs. The main function of the ES muscle is to hold the trunk in an upright position [30]. Due to the significant correlation, OWAS ACs seem to represent musculoskeletal load of the back well. This finding is supported by the results of Burdorf et al., who found a strong relationship between working time spent with a bent posture of the trunk and prevalence of back pain [38] and by the results of Kee at al., who observed a high association between working activities at an assembly line with a bent back and musculoskeletal injury of the back [39]. A small to medium significant correlation was found between muscle activity of trapezius muscles and OWAS ACs. No significant correlation was found between muscle activity of the deltoid muscles and OWAS ACs, which is caused by the high variance of muscle activity values in both OWAS ACs. These muscles are representative for a movement of the shoulder joint and the flexion of the arm [29]. Therefore, OWAS seems to be inappropriate for a representation of workload of the shoulder and arm region. Overall, OWAS shows varying degrees of predictive validity with regard to muscle activity, which ranges from small to high regarding the shoulder and arm region and the trunk region, respectively.

A small to medium significant correlation between muscle activity and assessment Zones of EN 1005-4 regarding the trunk (EN-T) were found in this study. Since EN-T focuses on workload of the trunk, we expected to find a large correlation between muscle activity of ES and EN-T. A possible explanation for the smaller than expected correlation between EN-T and muscle activity of ES could be the high variance of muscle activity values of ES in the different EN-T Zones, which are presented in Figs. 3 and 4. A small negative significant correspondence between muscle activity of the AD muscles and EN-T were found. Working postures with higher trunk inclinations are assigned to EN-T Zone 2 and EN-T Zone 3. With an increasing trunk inclination angle the relative height of the arm decreases even for greater shoulder angles. Since a reduction of arm height results in increasing blood flow and increasing oxygen supply of muscles in the arm [40] a reduction of arm height leads to a smaller muscle activity in the AD muscles in greater EN-T Zones. Overall, EN 1005–4 shows a variance of validity, which ranges from small to medium regarding the shoulder and arm region and the trunk region, respectively.

A medium to large significant correlation between muscle activity and assessment zones of EN 1005-4 regarding the shoulder (EN-S) was found by applying Spearman’s correlation analysis. The smallest correlation coefficient was found between muscle activity values of ES muscles and EN-S. A large correspondence was found between AD, UT and LT muscles and EN-S. Since EN-S focuses on workload of the shoulder and arm region this correspondence was expected. Overall, this large significant correspondence of muscle activity values of shoulder and arm muscles and EN-S Zones emphasises the high validity of assessment results of EN 1005-4 regarding the shoulder and arm region.

In general, there is a high variance of muscle activity values within the individual assessment classes (OWAS ACs and EN Zones). Thus, it remains questionable if a statement regarding workload due to working postures can be derived from such assessment results. A reclassification of assessment classes of OWAS and EN 1005-4 based on objective measurement of response variables of workload, i.e. muscle activity, is conceivable. For this purpose, it is necessary to introduce a threshold of muscle activity as a part of the guideline to classify working postures. Different publications state a muscle activity of 15% to 20% MVC as threshold of static working postures [26, 41, 42]. Such values are based on the interruption of blood flow resulting in an oxygen shortage of the muscle since the internal muscle pressure exceeds the blood pressure. Björkstén and Jonsson suggest a lower level of muscle activity of 5% to 6% MVC as a threshold for static working postures acceptable for a working time of one hour [43]. Thus, further studies are needed to specify a general threshold for a reclassification of assessment classes.

However, practitioners who are using observation-based assessment methods in practice need quick and easy to use methods [9] due to short time intervals of work processes in practise. Nevertheless, due to the constantly high number of MSDs [4], such requirements have to be reconsidered. For this reason, results of observation-based assessment methods, which are the basis for ergonomic improvements of workplaces in practise, seem to be insufficient for this purpose. Consequently, the validity of observation-based assessment methods should be revised using response variables. However, besides validity, studies should also focus on reliability, which has shown to be a problem of observation-based methods [9], and sampling strategy [44]. This study has contributed to the investigation of this problem because the validity of the two observation-based methods OWAS and EN 1005-4 has been investigated. In particular, OWAS shows potential for an improvement since data indicate only a small to medium predictive validity with regard to muscle activity. A consideration of interaction effects of body part postures by OWAS would facilitate an increase of validity since different studies [15], previous work of the authors [16] and also the results of this study showed the significance of interaction effects.

### 4.2. EMG amplitude

At first, significant effects of trunk inclination angle and shoulder flexion angle on muscle activity were found. Additionally, a significant interaction effect of trunk inclination angle and shoulder flexion angle on muscle activity was identified. These findings are supported by the results of Lim et al., who reported comparable results about interaction effects of trunk inclination and shoulder flexion [15]. Therefore, an interpretation of workload brought about by working postures has to be based on the postures of the different body parts. As our results show, interactions of different body part postures have a significant influence on muscle activity, which is a response variable and represents workload of working persons. The hypotheses regarding muscle activity stated in the introduction can be accepted based on the findings of our study.

### 4.3. Limitations

This study was conducted in a controlled laboratory setting. A generalising of our findings is possible only to a limited extent, since the here investigated cycle of static working posture and resting periods is not representative for work-rest cycles in practise. For instance, work in real work systems is oftentimes characterised by dynamic muscle contractions due to frequent changes of posture and multiple factors demanding external forces, for example handling of weights or mounting assembly parts, which were not considered in this investigation. Therefore, no conclusions can be drawn regarding validity of OWAS and EN 1005-4 for an investigation of dynamic muscle contractions. However, the observation-based methods are still based on the principle of work sampling with different sampling intervals [44, 45]. Consequently, it is not possible to evaluate the effects of dynamic muscle contractions on the musculoskeletal system using such observation-based methods, since only discontinuous data of postures are sampled [44]. To investigate dynamic muscle contractions for example the exposure variation analysis (EVA) introduced by Mathiassen and Winkel may be more appropriate [47]. Additionally, the low age profile of our sample group is not representative for all working persons in practise. Although the investigated hypotheses were supported by statistical evidence, the sample group is very small. However, even our sample size showed statistical significant effects. Thus, this study offers impetus for future investigations. Furthermore, for an investigation of static working postures based on EMG, an examination of skeletal muscles should be taken into account. However, during this study an investigation of skeletal muscles was not possible due to the necessity of invasive examination methods, e.g. needle electrodes. Regarding the investigation of dynamic working postures a measurement of further parameters like the compression force on the intervertebral disk at L5-S1 or energy expenditure measures like oxygen consumption and carbon dioxide may be considered [46]. However, since this study only covered static working postures, muscle activity represents the limiting factor of the human body [24, 25, 48]. Furthermore, the higher sensitivity of EN 1005-4 compared to OWAS stated in this work is limited to the working postures in the sagittal plane investigated in this study.

## 5. Conclusion

In conclusion, this study emphasises a need for further developments of observation-based assessment methods, since the two investigated methods OWAS and EN 1005–4 showed a variance of validity ranging from small to large regarding the assessment of static working postures. For an investigation of static working postures, EN 1005–4 is more recommendable to identify musculoskeletal injury risk of the shoulder and arm region, since this method showed a higher validity than OWAS. Additionally, this study revealed significant interaction effects of trunk inclination angle and shoulder flexion angle on muscle activity. To improve validity of observation-based methods it seems a possible way to further develop such methods by implementing a consideration of interaction effects into observation-based methods. Such improvements may increase not only validity of assessment results but also may form a better basis for the ergonomic improvement of working conditions in practise. The need for improvement of working conditions is emphasised by a constantly high prevalence of MSDs in the last decades.

## Conflict of interest

None to report.

## Funding

This article is part of the research project “ENgAge4Pro”, which was funded by the German Federal Ministry of Education and Research (BMBF, grant number 16SV6143). The authors would like to express their gratitude for the support they have received.

## References

[1] Widanarko B , Legg, S , Stevenson, M , Devereux J , Eng, A , Mannetje A ‘t et al. Gender differences in work-related risk factors associated with low back symptoms. Ergonomics. 2012;55(3):327–42. 22409170 10.1080/00140139.2011.642410

[2] Robert Koch-Institut. Gesundheit in Deutschland - die wichtigsten Entwicklungen. Gesundheitsberichterstattung des Bundes. English Title: Health in Germany – the most important developments. Federal health reporting. Berlin; 2016.

[3] Gallagher S , Schall MC . Musculoskeletal disorders as a fatigue failure process: Evidence, implications and research needs. Ergonomics. 2017;60(2):255–69. 27376409 10.1080/00140139.2016.1208848

[4] Vos T , Abajobir AA , Abate KH , Abbafati, C , Abbas KM , Abd-Allah F , et al. Global, regional, and national incidence, prevalence, and years lived with disability for diseases and injuries for 195 countries, 1990–2016: A systematic analysis for the Global Burden of Disease Study 2016. The Lancet. 2017;390(10100):1211–59. 10.1016/S0140-6736(17)32154-2 PMC5605509 28919117

[5] Winkel J , Mathiassen SE . Assessment of physical workload in epidemiologic studies: Concepts issues and operational considerations. Ergonomics. 1994;37(6):979–88. 8026455 10.1080/00140139408963711

[6] Roman-Liu D . Comparison of concepts in easy-to-use methods for MSD risk assessment. Appl Ergon. 2014;45(3):420–7. 23849897 10.1016/j.apergo.2013.05.010

[7] Kee D , Karwowski, W %. A comparison of three observational techniques for assessing postural loads in industry. Int J Occup Saf Ergon. 2007;13(1):3–14. 17362654 10.1080/10803548.2007.11076704

[8] Yazdani A , Wells, R %. Prevention of MSD within OHSMS/IMS: A systematic review of risk assessment strategies. WORK. 2012;41(Suppl 1):2765–7. 22317138 10.3233/WOR-2012-0522-2765

[9] Li G , Buckle, P %. Current techniques for assessing physical exposure to work-related musculoskeletal risks with emphasis on posture-based methods. Ergonomics. 1999;42(5):674–95. 10327891 10.1080/001401399185388

[10] David GC . Ergonomic methods for assessing exposure to risk factors for work-related musculoskeletal disorders. Occup Med (Lond). 2005;55(3):190–9. 15857898 10.1093/occmed/kqi082

[11] Takala E-P , Pehkonen, I , Forsman, M , Hansson G-A , Math-iassen SE , Neumann WP , et al. Systematic evaluation of observational methods assessing biomechanical exposures at work. Scandinavian Journal of Work, Environment & Health. 2010;36(1):3–24. 10.5271/sjweh.2876 19953213

[12] Karhu O , Kansi, P , Kuorinka, I %. Correcting working postures in industry: A practical method for analysis. Appl Ergon. 1977;8(4):199–201. 15677243 10.1016/0003-6870(77)90164-8

[13] McAtamney L , Corlett, E %. RULA: A survey method for the investigation of work-related upper limb disorders. Appl Ergon. 1993;24(2):91–9. 15676903 10.1016/0003-6870(93)90080-s

[14] Hignett S , McAtamney, L %. Rapid Entire Body Assessment (REBA). Appl Ergon. 2000;31(2):201–5. 10711982 10.1016/s0003-6870(99)00039-3

[15] Lim C-M , Jung M-C , Kong Y-K . Evaluation of upper-limb body postures based on the effects of back and shoulder flexion angles on subjective discomfort ratings heart rates and muscle activities. Ergonomics. 2011;54(9): 849–57. 21943119 10.1080/00140139.2011.600777

[16] Hellig T , Mertens, A , Brandl, C %. The interaction effect of working postures on muscle activity and subjective discomfort during static working postures and its correlation with OWAS. International Journal of Industrial Ergonomics. 2018;68:25–25.

[17] Denis D , Lortie, M , Rossignol, M %. Observation procedures characterizing occupational physical activities: Critical review. Int J Occup Saf Ergon. 2000;6(4):463–91. 11135680 10.1080/10803548.2000.11076467

[18] Brandl C , Mertens, A , Schlick CM . Ergonomic analysis of working postures using OWAS in semi-trailer assembly, applying an individual sampling strategy. Int J Occup Saf Ergon. 2017;23(1):110–7. 27192477 10.1080/10803548.2016.1191224

[19] Kayis B , Kothiyal, K %. A Multilevel Approach to Manual Lifting in Manufacturing Industries. Int J Occup Saf Ergon. 1996;2(3):251–61. 10602590 10.1080/10803548.1996.11076353

[20] Olendorf MR , Drury CG . Postural discomfort and perceived exertion in standardized box-holding postures. Ergonomics. 2001;44(15):1341–67. 11936827 10.1080/00140130110085358

[21] Burdorf A , Derksen, J , Naaktgeboren, B , van Riel M . Measurement of trunk bending during work by direct observation and continuous measurement. Appl Ergon. 1992;23(4):263–7. 15676874 10.1016/0003-6870(92)90154-n

[22] van der Beek AJ , Erik Mathiassen S , Windhorst, J , Burdorf, A %. An evaluation of methods assessing the physical demands of manual lifting in scaffolding. Appl Ergon. 2005;36(2):213–22. 15694076 10.1016/j.apergo.2004.10.012

[23] Brandl C . Ergonomische Analyse von Körperhaltungen in Produktionssystemen für eine computergestützte Arbeitsgestaltung und -organisation. English Title: Ergonomic Analysis of Working Postures in Production Systems for Computer-Aided Industrial Engineering. [Dissertation]. Aachen: RWTH Aachen; 2017.

[24] Rohmert W. Ergonomics: Concept of work, stress and strain. AppliedPsychology. 1986; 35(2):159–80.

[25] Rohmert W . Formen menschlicher Arbeit. English Title: Types of human work In: Rohmert W , Rutenfranz, J , English Title: Practical work physiology. 3. Auflage. Stuttgart: Thieme Verlag; 1983 pp.5–29.

[26] Rohmert W . Untersuchungen über Muskelermüdung und Arbeitsgestaltung. English Title: Investigations on muscle fatigue and work design [Post-doctoral thesis]. Aachen: RWTH Aachen; 1962.

[27] Schlick C , Luczak, H , Bruder, R. Arbeitswissenschaft. English Title: Ergonomics. Heidelberg Springer; 2010.

[28] Srinivasan D , Sinden KE , Mathiassen SE , Côté JN . Gender differences in fatigability and muscle activity responses to a short-cycle repetitive task. Eur J Appl Physiol. 2016;116(11-12):2357–65. 27743025 10.1007/s00421-016-3487-7 PMC5118407

[29] Perotto A , Delagi EF . Anatomical guide for the electromyo-grapher: The limbs and trunk 4th Springfield, IL Charles C Thomas; 2005.

[30] Hermens HJ . European recommendations for surface Elec-troMyoGraphy: Results of the SENIAM project. Enschede: Roessingh Research and Development; 1999 (SENIAM; Vol 8).

[31] Schünke M , Schulte, E , Schumacher, U %. Prometheus. LernAtlas der Anatomie. Band 1, Allgemeine Anatomie und Bewegungssystem. English Title: Promethues. Learning Atlas of Anatomy. Volume 1, General Anatomy and Movement System. 4. überarbeitete und erweiterte Auflage; 2014

[32] Burden A . How should we normalize electromyograms obtained from healthy participants? What we have learned from over 25 years of research. J Electromyogr Kinesiol. 2010;20(6):1023–35. 10.1016/j.jelekin.2010.07.004 20702112

[33] Mathiassen SE , Winkel, J , Hagg GM . Normalization of surface EMG amplitude from the upper trapezius muscl ergonomic studies - A review. J Electromyogr Kinesiol. 1995;5(4):197–226. 20719652 10.1016/1050-6411(94)00014-x

[34] Dahlqvist C , Nordander, C , Granqvist, L , Forsman, M , Hansson G-Å . Comparing two methods to record maximal voluntary contractions and different electrode positions in recordings of forearm extensor muscle activity: Refining risk assessments for work-related wrist disorders. Work. 2018;59(2):231–42. 29355119 10.3233/WOR-172668 PMC5870034

[35] Patrizi A , Pennestrì, E , Valentini PP %. Comparison between low-cost marker-less and high-end marker-based motion capture systems for the computer-aided assessment of working ergonomics. Ergonomics. 2016;59(1):155–62. 26043178 10.1080/00140139.2015.1057238

[36] Braganca S , Arezes, P , Carvalho, M , Ashdown SP , Castel-lucci I , Leão, C %. A comparison of manual anthropometric measurements with Kinect-based scanned measurements in terms of precision and reliability. Work. 2018;59(3):325–39. 29630575 10.3233/WOR-182684

[37] Cohen J . A power primer. Psychol Bull. 1992;112(1):155–9. 19565683 10.1037//0033-2909.112.1.155

[38] Burdorf A , Govaert, G , Elders, L %. Postural load and back pain of workers in the manufacturing of prefabricated concrete elements. Ergonomics. 1991;34(7):909–18. 1833181 10.1080/00140139108964834

[39] Kee D , Kamalinia, M , Oliaee, M , Daneshmandi, H , Mohammadi, H %. An ergonomic intervention to relieve mus-culoskeletal symptoms of assembly line workers at an electronic parts manufacturer in Iran. Work. 2018. 10.3233/WOR-182822 30475781

[40] Lin C-L , Wang M-JJ , Drury CG , Chen Y-S . Evaluation of perceived discomfort in repetitive arm reaching and holding tasks. International Journal of Industrial Ergonomics. 2010;40(1):90–6.

[41] Rohmert W. Statische Arbeit. English Title: Static work In: Rohmert W , Rutenfranz, J , Praktische Arbeitsphysiologie English Title: Practical work physiology. 3. Auflage. Stuttgart: Thieme Verlag; 1983 pp. 34–43.

[42] Kroemer KHE . Cumulative trauma disorders: Their recognition and ergonomics measures to avoid them. Appl Ergon. 1989;20(4):274–80. 15676745 10.1016/0003-6870(89)90190-7

[43] Björkstén M , Jonsson, B %. Endurance limit of force in long-term intermittent static contractions. Scand J Work Environ Health. 1977;3(1):23–7. 847427 10.5271/sjweh.2795

[44] Brandl C , Mertens, A , Schlick CM . Effect of sampling interval on the reliability of ergonomic analysis using the Ovako working posture analysing system (OWAS). International Journal of Industrial Ergonomics. 2017;57:68–68.

[45] Louhevaara V , Suurnäkki, T %. OWAS: A method for the evaluation of postural load during work: Institute of Occupational Health. Centre for Occupational Safety; 1992.

[46] Levine JA . Measurement of energy expenditure. PHN. 2005;8(7a):79. 10.1079/phn2005800 16277824

[47] Mathiassen SE , Winkel, J %. Quantifying variation in physical load using exposure-vs-time data. Ergonomics. 1991;34(12):1455–68. 1800110 10.1080/00140139108964889

[48] Kirchner J-H . Belastungen und Beanspruchungen — Einige begriffliche Klärungen zum Belastungs- Beanspruchungs-Konzept In: Hackstein R , Heeg F-J , Below F von , Arbeitsorganisation und Neue Technologien. Berlin, Heidelberg: Springer Berlin Heidelberg; 1986 pp. 553–69.

